# ADHD-related symptoms and attention profiles in the unaffected siblings of probands with autism spectrum disorder: focus on the subtypes of autism and Asperger’s disorder

**DOI:** 10.1186/s13229-017-0153-9

**Published:** 2017-07-25

**Authors:** Yi-Ling Chien, Miao-Chun Chou, Yen-Nan Chiu, Wen-Jiun Chou, Yu-Yu Wu, Wen-Che Tsai, Susan Shur-Fen Gau

**Affiliations:** 1 Department of Psychiatry, National Taiwan University Hospital and College of Medicine, No.7, Chung-Shan South Road, Taipei, 10002 Taiwan; 2 Graduate Institute of Clinical Medicine, College of Medicine, National Taiwan University, Taipei, Taiwan; 3 Department of Child and Adolescent Psychiatry, Chang Gung Memorial Hospital, Kaohsiung Medical Center, Chang Gung University College of Medicine, Kaohsiung City, Taiwan; 4 Department of Psychiatry, Chang Gung Memorial Hospital- Linkou Medical Center, Chang Gung University College of Medicine, Taoyuan, Taiwan

**Keywords:** Autism spectrum disorder, Sibling, Attention, Continuous performance test, Endophenotype

## Abstract

**Background:**

The presence of attention-deficit/hyperactive disorder (ADHD) symptoms and impaired attention performance are commonly noted in individuals with autism spectrum disorder (ASD). However, little is known about attention performance in their unaffected siblings. This study aimed to investigate the ADHD-related traits and attention performance in unaffected siblings of probands with autism and Asperger syndrome (AS), as well as the clinical correlates of ADHD-related traits.

**Methods:**

We assessed the intention, hyperactivity-impulsivity, and oppositional symptoms, and attention profiles of 199 probands with a diagnosis of ASD (122 autism, 77 AS), their unaffected siblings, and 196 typically developing controls (TD) by their parents’ reports on the ADHD-related symptoms and the Connors' Continuous Performance Test (CCPT), respectively.

**Results:**

Compared to TD, unaffected siblings of ASD probands were more hyperactive/impulsive and oppositional, particularly unaffected siblings of AS probands. In CCPT, unaffected siblings of AS have intermediate levels of performance between probands with AS and TD on focused attention and sustained attention but were not statistically different from AS probands or TD in these attention profiles. In contrast, unaffected siblings of autism probands have significantly better CCPT performance when compared to autism probands but not to TD. In addition, stereotyped behaviors predicted ADHD-related traits in both sibling groups, but distinctive patterns of other correlates for ADHD-related traits were found between the two sibling groups.

**Conclusions:**

This work suggested that unaffected siblings of AS, but not autism, have more hyperactive/impulsive traits and a trend of pervasive attention deficits assessed by CCPT which might serve as potential endophenotypes for genetic studies in AS.

**Trial registration:**

ClinicalTrials.gov, NCT01582256

**Electronic supplementary material:**

The online version of this article (doi:10.1186/s13229-017-0153-9) contains supplementary material, which is available to authorized users.

## Background

The heritability of ASD has been estimated higher than 90% [1], with sibling recurrent risk ratio around 3~18.7% [2, 3], indicating that genetic component plays an essential role in the pathogenesis of ASD [4, 5]. Despite high heritability, numerous genetic studies cannot converge to consistent results, mainly because of its complex phenotypic and genetic heterogeneity. Endophenotype approach has been proposed in searching for genetic etiology of complex diseases including ASD [6–8] to increase the power to localize and identify disease-related quantitative trait loci than behavioral phenotype approach [9]. Endophenotypes can be defined as measurable biomarkers that are heritable, state-independent, associated with the disease and co-segregated with the disease within the family, and show a higher rate in “unaffected” family members than in general population. Through identifying the “disease-related quantitative traits” in the unaffected siblings, future genetic studies in ASD may target at a more objective/specific phenotype to search for the disease-related genes.

Attention deficits are commonly associated manifestations of ASD. Literature documents that 52–78% of individuals with ASD also meet the diagnostic criteria for attention-deficit/hyperactivity disorder (ADHD) [10–15]. Oppositional defiant disorder (ODD), though relatively less studied, was also more prevalent in youth with ASD than in controls [16, 17]. These symptoms may persist into adolescence [12], implying that ADHD-related symptoms could be a trait rather than state phenomenon. Individuals with ASD not only showed a higher prevalence of ADHD-related symptoms [13, 15–21] but also demonstrated worse performance on the attention tasks such as the Continuous Performance Test (CPT) [22, 23]. These impaired CPT performance included higher variability in reaction time [24–26] as well as more omission errors and poorer sustained attention [27]. Consistent with Western research [13, 15, 18–21, 24–26], our previous study also showed that youths with ASD had more inattentive, hyperactive/impulsive, and oppositional symptoms, and also showed worse focused attention, cognitive impulsivity, and vigilance assessed by CPT compared to age-matched TD controls [27]. A recent study used a large Finnish ASD cohort to demonstrate that siblings of probands with ASD were at risk for ADHD (5.3% in siblings vs. 1.5% in controls, adjusted relative risk 3.7), and conduct and oppositional disorders (5.0 vs. 1.9%, adjusted relative risk 2.8) [28]. However, 10.5% of these siblings were diagnosed with ASD. A Swedish register-based cohort study suggested that relatives of individuals with ASD were at a higher risk for ADHD compared with relatives of individuals without ASD [29]. Nevertheless, whether the “unaffected siblings” of probands with ASD share the similar ADHD-related symptoms and attention deficits has not been well explored yet.

Some studies have shown that attention deficits on CPT [30, 31] can be an endophenotype for ADHD [32–35]. The heritability of sustained attention is about 60% [36]. Evidence suggests that sustained attention may be useful in detecting the genetic effects underlying several complex disorders [30, 33]. Several genetic studies used CPT deficit as an intermediate phenotype and reported significant associations with candidate genes in ADHD [37–39]. For example, a recent study showed that the genetic variants of *N*-methyl-d-aspartate receptor subunit-encoding genes (GRIN2A and GRIN2B) confer an increased susceptibility to attentional impairment measured by CPT in ADHD patients [40]; a genome-wide association study of CPT performance in adults with ADHD reported 27 suggestive loci were associated with CPT outcomes [41], implying that CPT deficits might be helpful in detecting genetic etiology of ADHD. In contrast, despite evidence of impaired sustained attention in ASD as shown in ADHD [42], there have been no studies testing whether attention profiles assessed by CPT can be an intermediate phenotype or endophenotype for ASD. Several studies have shown that the unaffected siblings, like ASD probands, revealed executive dysfunction including impaired cognitive flexibility [43–45], generativity/ideational fluency [45, 46], response inhibition [45], and planning [43, 47, 48]. These results suggest that the above deficits could be strong candidate endophenotypes for ASD. Some researchers proposed that these endophenotypes can help delineate subgroups from more homogeneous etiologies, allowing the clinicians to develop more specific interventions [45]. As CPT is a widely used neuropsychological test with a relatively simple paradigm examining a fundamental neurocognitive function, it is clinically relevant to test whether attention deficits measured by CPT can be an endophenotype for ASD.

The previous study suggested different profiles of ADHD symptoms and Conners’ CPT (CCPT) performance between autistic disorder and Asperger syndrome (AS). One of the specific findings is that youths with AS demonstrated more oppositional symptoms, worse sustained attention but fewer omission errors and longer reaction time on CCPT than youths with autistic disorder [27]. Although the diagnosis of AS no longer existed in DSM-5, these ASD subgroups may still be distinct from one another across other features central to the conceptualization of ASD [49, 50]. Studies exploring the differences on cognitive profiles [51] or emotion recognition test [52] continued to report quantitative and qualitative differences between the subgroups and suggested to find ways to meaningfully classify ASD in clinical practice and research [53, 54]. It is unclear whether the unaffected siblings of autism proband and AS proband both showed ADHD-related traits and CCPT deficits relative to TD. In addition, the clinical correlates of ADHD-related traits are another focus of interest. ADHD symptoms were shown to be associated with CCPT deficits in individuals with ADHD [55]. Our prior finding of low-grade correlations between ADHD-related symptoms and CCPT deficits in youths with ASD, not as remarkable as in ADHD [27], suggested that the presentation of ADHD traits in ASD probands may not be related to the same neurocognitive impairment observed in ADHD. Instead, ADHD symptoms in youths with ASD had been shown to be associated with autistic symptoms [13]. Whether these phenomena also exist in unaffected siblings of ASD probands waits to be elucidated. Sokolova et al. recently explored the relationship between ASD and ADHD symptoms by applying causal modeling and found distinct pathways between inattention and social cognition, and between hyperactivity and stereotypy in a sample mixed with probands with ASD, ADHD, their unaffected siblings, and controls [56]. Whether the associations between ADHD-related traits and autistic core symptoms are different in unaffected siblings of autism and those of AS probands are of particular interest. It is clinically relevant to use model selection to identify the most important correlates among the potential correlates (i.e., autistic symptom subscores and CCPT indexes) for ADHD-related traits.

This study aimed to investigate the ADHD-related traits and CCPT performance in the unaffected siblings of ASD probands, and the clinical correlates (i.e., CCPT deficits and autistic symptoms) of ADHD-related traits. The analyses were conducted separately in autistic disorder (autism) and AS to examine whether ADHD-related traits and attention deficits assessed by the CCPT deficits were expressed differently in autism and AS. We hypothesized that the unaffected siblings, like probands, may have more ADHD-related symptoms and worse CCPT performance compared to TD. Also, the ADHD-related traits might be associated with autistic traits and CCPT performance differently in unaffected siblings of autism probands and those of AS probands.

## Methods

### Participants and procedures

The sample consisted of 122 probands with autism (male, 88.5%; age range, 6–16 years; mean age, 10.4±2.5 years), 122 unaffected siblings of autism probands (male, 50.5%; age range, 6–18 years; 10.6±3.2 years), 77 probands with AS (male, 90.9%; age range, 6–18 years; age range, 11.5±2.9 years), 77 unaffected siblings of AS probands (male, 49.4%; age range, 6–17 years; mean age, 10.7±3.0 years), and 196 TD (male, 65.8%; age range, 7–18 years; mean age, 11.1±2.9 years). The probands are all Han Chinese recruited from the outpatient clinic of Psychiatric Department from National Taiwan University Hospital and schools in Northern Taiwan. The TD youths, also Han Chinese, were recruited at schools in the same districts of the ASD groups through the teachers and principals rather than through an advertisement. The clinical diagnoses of all the participants were made by senior board-certificated child psychiatrists according to the DSM-IV diagnostic criteria for autistic disorder (autism) or Asperger’s disorder (AS), and were further confirmed by using the Chinese version of the Autism Diagnostic Interview-Revised (ADI-R) [57] for all the probands and some of siblings and TD if a diagnosis of ASD was suspected; the Chinese version of the Kiddie epidemiologic version of the schedule for affective disorders and Schizophrenia (K-SADS-E) for all the participants to screen for any current and previous mental disorders such as ADHD, schizophrenia, mood disorders, anxiety disorders, or developmental disorders [58]. Three youths with TD were excluded, resulting in a total of 199 pairs of probands and unaffected siblings, and 196 age-matched TD. The Research Ethics Committee of National Taiwan University Hospital approved this study before implementation. After the purposes and procedures of the study were fully explained and confidentiality was assured, written informed consent was obtained from the participants and their parents. The participants were then assessed with the CCPT and the *Wechsler Intelligence Scale for Children—Fourth Edition* [59] to ensure that all participants have full-scale IQ above 70 and can understand the procedure of the tasks; their parents received the ADI-R interview and reported the participants’ autistic and ADHD-related traits.

### Measures

#### The Conners’ Continuous Performance Test for Windows II (CCPT) [60]

The CCPT is a widely used computerized task to assess attention performance by non-X type CPT test of go/no-go paradigm. The 360 trials, composed of 10% no-go targets, were presented with six blocks and three sub-blocks (20 trials in each sub-block). The sub-blocks were different in inter-stimulus intervals (ISIs) as 1, 2, and 4 s, and the sequences of ISIs are randomly organized. There are 12 indexes covering different domains of attention: (1) omission errors: the number of times not responding to a target; (2) commission errors: the number of times responding to a non-target; (3) reaction time (RT); (4) variability: intra-individual variability in RT; (5) perseveration: a RT less than 100 ms; (6) detectability: the ability to discriminate between targets and non-targets; (7) hit RT standard errors (hit RT SE); (8) response style; (9) hit RT block change; (10) hit RT standard errors block change (hit RT SE block change); (11) hit RT ISI change; (12) hit RT standard error ISI change (hit RT SE ISI change).

This study employed the 4-factor structure proposed by Egeland and Kovalik-Gran [61]: (1) focused attention: omission errors, RT variability, hit RT SE, and perseverations; (2) cognitive impulsivity: commission errors, hit RT, and response style; (3) sustained attention: hit RT and hit RT SE changed across blocks; and (4) vigilance: hit RT and hit RT SE changed across different ISIs.

#### ADI-R

The ADI-R [62] is a standardized, comprehensive, semi-structured, investigator-based interview with the caregivers. It covers most developmental and behavioral aspects of ASD, including reciprocal social interaction, communication, and repetitive behaviors and stereotyped patterns, for children with a mental age from 18 months into adulthood. The ratings were based on an assessment of the current condition and the most severe state at 4–5 years old recalled by the caregivers. The Chinese ADI-R was approved by the World Psychological Association in 2007 and has been widely used for assisting the clinical diagnosis of ASD [63].

#### The Chinese version of the Swanson, Nolan, and Pelham, version IV scale (SNAP-IV)

The parents reported the ADHD and ODD symptoms on the SNAP-IV [64], which is a 26-item scale rating on a 4-point Likert scale with a score of 0 for “not at all,” 1 for “just a little,” 2 for “quite a bit,” and 3 for “very much.” There are 18 items parallel to the core symptoms of DSM-IV ADHD (items 1–9 for inattention symptoms; items 10–18 for hyperactivity/impulsivity symptoms), and eight items based on the DSM-IV of ODD symptoms. The psychometric properties and norm of the Chinese SNAP IV-Parent form have been established in Taiwan [65] showing the same 3-factor structure as its English version. It has been used in many clinic-based and community-based studies in Taiwan (e.g., [66–70]). The internal consistency of SNAP-IV subscores was excellent in this study (Cronbach’s alpha ≥ 0.9).

#### The Chinese Version of the Social Communication Questionnaire (SCQ)

The SCQ is a parent-report questionnaire to screen for autistic symptoms for individuals older than 4 years. It contains 40 yes-or-no items and corresponds to the three core symptoms of autism, i.e., impairment in social development, communication, and stereotyped behaviors and restricted interests that parallel to the ADI-R. The scores of SCQ are substantially unaffected by age, gender, language level, and performance IQ. The Chinese SCQ has satisfactory reliability and validity [57] and has been used in ASD research in Taiwan. The internal consistency of SCQ subscores was good to excellent in this study (Cronbach’s alpha ≥ 0.8).

#### The Chinese version of the Child Behavior Checklist (CBCL)

The CBCL [71] is a parent-report questionnaire to screen for broad spectrum behavior symptoms in youths aged 4–18. It includes eight constructs, i.e., withdrawn, somatic complaints, anxious/depressed, social problems, thought problems, attention problems, delinquent behavior, and aggressive behaviors. Items were rated on a 3-point scale from 0 (not true) to 2 (very true or often true). The Chinese CBCL has been widely used to measure behavioral syndromes in Taiwanese child and adolescent populations [72]. The subscore of “attention problems” was chosen for group comparison in this study to measure both inattentive and hyperactivity symptoms. The internal consistency of this subscore was good in this study (Cronbach’s alpha = 0.89).

### Statistical analysis

The SAS program 9.2 (SAS Institute Inc, Cary NC, USA) was used for data analysis. We compared ADHD-related traits (inattentive, hyperactive/impulsive, and oppositional) and CCPT indexes between “ASD probands” (autism probands + AS probands), “unaffected siblings” (of autism probands and AS probands), and “TD” first. Then, we performed three-group comparisons in “autism subgroups” (autism probands, unaffected siblings of autism, and TD) and in “AS subgroups” (AS probands, unaffected siblings of AS, and TD) separately. In these comparisons, probands and their siblings were compared to the whole TD controls as the same reference group. For these three group comparisons, we used the mixed model (PROC MIXED procedure) with random effects to address the lack of independence within the same family to compare the means of IQ, ADHD-related traits, and each CCPT index between probands, unaffected siblings, and TD; sex and age were controlled in the models. The multiple comparisons in the three-group comparisons were corrected by Bonferroni method. The multiple comparisons for the four ADHD-related trait subscores and 12 CCPT indexes were corrected by false discovery rate for that the variables were not totally independent from each other and Bonferroni correction may be too stringent. A trend test was applied for testing the linear trend among probands with AS, their unaffected siblings, and TD for CCPT performance. Effect size, presented by Cohen’s *d*, was also calculated to show the magnitude of group differences in ADHD-related traits.

Finally, to examine the correlates of ADHD-related symptoms in the unaffected siblings, we selected significant correlates from age, sex, autistic traits (social deficits, communication deficits, and stereotyped behaviors on the SCQ), and CCPT indexes for each of inattentive, hyperactive/impulsive and oppositional traits by a multivariate model with backward eliminating method, and presented the parameters of model fitting. Siblings of autism probands and AS probands were analyzed separately. The significance level was set at *p* < 0.05 level.

## Results

### Sample characteristics

Table 1 presents the demographic data of two ASD groups, two unaffected sibling groups of autism or AS probands, and TD groups. The two ASD groups and TD group were male predominant, while half of the unaffected sibling groups were females. There was no significant difference in age distribution among the five groups (Table 1). Probands with autism had significantly lower IQ compared to their unaffected siblings and TD; probands with AS had lower IQ only in performance IQ than TD while the unaffected siblings of AS probands were not different from AS probands and TD (Table 1). Both proband groups had more autistic symptoms than their unaffected siblings and TD assessed by the Chinese SCQ (see Additional file 1).

**Table 1:** Demographic data and IQ profiles of probands, unaffected siblings (US), and typically developing controls (TD)

Group	Autism(*n* = 122)	AS(*n* = 77)	US of autism(*n* = 122)	US of AS(*n* = 77)	TD(*n* = 196)		
						*x* ^*2*^, *F*	*P*
Male (%)	108 (88.5%)	70 (90.9%)	61 (50.0%)	38 (49.4%)	129 (65.8%)	73.49	<.001
Age: mean ± SD (age range)	10.4 ± 2.5 (6.0–16.0)	11.5 ± 2.9 (6.0–18.0)	10.6 ± 3.2 (6.0–18.0)	10.7 ± 3.0 (6.0–17.0)	11.1 ± 2.9 (7.0–18.0)	2.25	0.063
IQ profiles							
Verbal IQ	88.18 ± 23.82	109.04 ± 12.78	105.36 ± 14.38	111.03 ± 9.70	112.13 ± 11.18	44.77	<.001
Performance IQ	95.63 ± 20.62	105.68 ± 15.17	107.39 ± 15.26	106.76 ± 12.81	110.89 ± 13.04	17.48	<.001
Full-scale IQ	91.11 ± 21.83	107.97 ± 12.86	106.61 ± 14.44	109.57 ± 10.41	112.44 ± 11.37	36.85	<.001

### ADHD and oppositional traits

When autism and AS were lumped together for three group comparison, probands had significantly higher ADHD and oppositional traits than unaffected siblings and TD, while unaffected siblings were only different from TD by more severe oppositional trait (see Additional file 2). The same pattern was noted when autism and AS were analyzed separately, yet the unaffected siblings of AS probands not only showed more oppositional trait but also displayed higher hyperactive/impulsive trait than TD (Table 2).

**Table 2:** Comparison of ADHD and oppositional symptoms between probands, unaffected siblings (US), and TD

	Autism (*n* = 122)	US of autism (*n* = 122)	TD (*n* = 196)				Cohen’s *d*		
				*F*	*P*	Comparison^a^	A:TD	US:TD	A:US
SNAP-IV									
Inattentive	15.77 ± 6.50	6.34 ± 5.65	5.46 ± 4.65	114.96	<.001	A>US,TD	1.82	0.17	1.55
Hyperactive/impulsive	10.60 ± 7.04	3.77 ± 4.58	2.92 ± 3.93	69.69	<.001	A>US,TD	1.35	0.20	1.15
Oppositional	8.36 ± 5.75	5.98 ± 4.73	3.60 ± 3.89	30.17	<.001	A>US>TD	0.97	0.55	0.45
CBCL									
Attention problems	10.60 ± 4.35	3.55 ± 3.61	2.83 ± 2.91	162.70	<.001	A>US,TD	2.10	0.22	1.76
	AS (*n* = 77)	US of AS (*n* = 77)	TD (*n* = 196)				Cohen’s *d*		
				F	*P*	Comparison^a^	AS:TD	US:TD	AS:US
SNAP-IV									
Inattentive	15.88 ± 6.72	6.58 ± 6.55	5.46 ± 4.65	75.39	<.001	AS>US,TD	1.80	0.20	1.40
Hyperactive/impulsive	11.83 ± 6.89	4.61 ± 5.47	2.92 ± 3.93	65.12	<.001	AS>US>TD	1.59	0.35	1.16
Oppositional	11.61 ± 6.21	6.64 ± 6.02	3.60 ± 3.89	53.62	<.001	AS>US>TD	1.55	0.60	0.81
CBCL									
Attention problems	11.82 ± 4.51	3.96 ± 4.21	2.83 ± 2.91	138.04	<.001	AS>US,TD	2.37	0.31	1.80

All passed false discovery date correction *p* < 0.05

*Abbreviations*: *CBCL* the Child Behavior Checklist, *SNAP-IV* the Swanson, Nolan, and Pelham, version IV scale

^a^Bonferroni correction *p* < 0.05

The differences between probands and TD showed large effect sizes (defined by Cohen’s *d* > 0.8) on the inattentive trait (AS probands 1.80 vs. autism probands 1.82), hyperactive/impulsive trait (1.59 vs. 1.35), oppositional behaviors (1.55 vs. 0.97) on SNAP-IV, and CBCL attention problems (2.37 vs. 2.10). In contrast, the effect sizes of the differences between unaffected siblings and TD were small (Cohen’s *d* > 0.2) [i.e., SNAP-IV hyperactive/impulsive trait, CBCL attention problems] to medium (Cohen’s *d* > 0.5) [i.e., SNAP-IV oppositional trait], or even lower [i.e., *Cohen’s d* 0.17 for SNAP-IV inattentive trait in autism probands] (Table 2).

### Attention profiles among ASD, unaffected siblings, and TD

Probands with ASD (autism and AS) performed poorer than unaffected siblings and TD on most CCPT indexes, while the attention profiles of the unaffected siblings were not different from those of TD except a longer RT in unaffected siblings than TD (see Additional file 2).

### Attention profiles among autism, unaffected siblings, and TD

When we compared autism probands, their unaffected siblings, and TD, autism probands performed worse than unaffected siblings and TD on all five indexes of focused attention (i.e., omission errors, hit RT SE, variability, perseveration, and detectability) and two indexes of cognitive impulsivity (i.e., commission and reaction time), without significant differences between unaffected siblings of autism probands and TD (see Additional file 3).

### Attention profiles among AS, unaffected siblings, and TD

When we compared AS probands, their unaffected siblings, and TD, AS probands performed worse than TD on focused attention (i.e., omission errors, hit RT SE, and variability) and sustained attention (hit SE block change) (Table 3); unaffected siblings of AS were in the intermediate position without significant difference from either TD or AS probands on focused attention (i.e., omission errors, hit RT SE, and variability) and sustained attention (hit SE block change) (see Additional file 4). Trend tests were significant in most indexes of focused attention, sustained attention, and vigilance (i.e., hit RT ISI) (Table 3).

**Table 3:** Comparison of CCPT performance between AS, unaffected siblings (US) of AS probands, and TD

	AS (*n* = 77)	US of AS (*n* = 77)	TD (*n* = 196)				Trend test	
				*F*	*p*	Comparison^a^	*F*	*p*
Focused attention								
Omission	10.75±19.13	8.34±9.18	6.35±7.41	5.33	0.007*	AS>TD	9.09	0.003**
RT SE	10.73±7.42	10.11±6.58	8.63±5.50	7.25	0.001*	AS>TD	14.60	0.000**
Variability	23.33±25.53	19.99±17.71	16.89±16.54	4.91	0.010*	AS>TD	11.91	0.001**
Perseveration	9.26±13.78	9.31±16.57	7.02±14.31	2.00	0.142	–	7.50	0.007**
Detectability	0.37±0.37	0.44±0.35	0.43±0.36	1.35	0.266	–	0.68	0.411
Cognitive impulsivity								
Commission	22.22±8.56	20.82±8.20	20.85±8.31	1.85	0.165	–	1.23	0.269
Reaction time	396.03±89.55	399.66±83.98	381.04±67.13	4.91	0.010*	AS>TD	4.25	0.040**
Response style	0.60±0.43	0.55±0.34	0.58±1.25	0.62	0.541	–	2.88	0.091
Sustained attention								
Hit RT block change	0.01±0.04	0.01±0.04	0.01±0.03	2.44	0.094	–	4.82	0.029**
Hit SE block change	0.09±0.12	0.06±0.13	0.04±0.09	4.59	0.013*	AS>TD	6.80	0.010**
Vigilance								
Hit RT ISI change	0.09±0.05	0.08±0.05	0.08±0.04	3.15	0.049	AS>TD	5.44	0.020**
Hit SE ISI change	0.13±0.18	0.12±0.17	0.10±0.16	1.56	0.218	–	2.23	0.136

*Abbreviations*: *CCPT* Conners’ Continuous Performance Test; *RT* Reaction time; *β* estimate of regression coefficient; *SE* standard error, *ISI* inter-stimulus interval

*False discovery rate-adjusted *p* < 0.05

**Trend test *p* < 0.05

^a^Bonferroni correction *p* < 0.05

### Correlates for ADHD-related traits in unaffected siblings

Table 4 presents the regression coefficient estimates in the final models for ADHD-related traits in unaffected siblings. In general, stereotyped symptoms were positively associated with inattentive, hyperactive/impulsive, and oppositional traits for both unaffected siblings groups. For inattentive trait, social deficits and “perseveration” on the CCPT were selected in the model for unaffected siblings of autism, while response style was selected for unaffected siblings of AS. For hyperactive/impulsive trait, perseveration was included in the model for unaffected siblings of autism, while age and communication deficits were included in the model for unaffected siblings of AS. The oppositional traits were associated with detectability and hit SE block change of CCPT in unaffected siblings of AS. Notably, the models for ADHD-related traits had better model fitting in unaffected siblings of AS (*R*-square 0.428~0.559) than unaffected siblings of autism (*R*-square 0.094~0.187) (Table 4).

**Table 4:** Model fitting for autistic symptoms and CCPT performance to predict ADHD symptoms in unaffected siblings (US) of autism probands and in US of AS probands

	Inattentive				Hyperactive/impulsive				Oppositional			
	US of autism		US of AS		US of autism		US of AS		US of autism		US of AS	
	*β* ± SE	*p*	*β* ± SE	*p*	*β* ± SE	*p*	*β* ± SE	*p*	*β* ± SE	*p*	*β* ± SE	*p*
Age							−0.30 ± 0.14	0.038				
Autistic symptoms (SCQ)												
Social deficits	0.43 ± 0.18	0.017										
Communication deficits							0.89 ± 0.44	0.047				
Stereotyped behaviors	1.20 ± 0.37	0.002	1.99 ± 0.28	<.001	0.71 ± 0.31	0.024	1.41 ± 0.031	<.001	1.13 ± 0.32	<.001	1.87 ± 0.26	<.001
CCPT^a^												
Perseveration	0.14 ± 0.05	0.003			0.13 ± 0.04	0.001						
Detectability											−4.36 ± 1.67	0.011
Response style			3.51 ± 1.67	0.039								
Hit SE block change											−10.20 ± 4.44	0.025
Model fitting												
*R*-square (%)	9.4		48.7		11.6		55.9		18.7		42.8	
*F* value	12.44		35.16		7.77		30.84		9.01		18.20	
*P* value	<.001		<.001		<.001		<.001		<.001		<.001	

There was no effect from sex or parental educational level

*Abbreviations*: *SCQ* the Social Communication Questionnaire, *CCPT* Conner’s Continuous Performance Test, *RT* reaction time, *β* estimate of regression coefficient, *SE* standard error, *ISI* inter-stimulus interval

^a^Only the significant CCPT indexes were presented

## Discussion

To the best of our knowledge, the current investigation is the first study to combine the clinical assessment of ADHD-related traits and neuropsychological assessment of attention performance by the CCPT in unaffected siblings of probands with ASD. Like previous studies [10–15, 24–26], we found probands with ASD had more severe inattentive, hyperactive/impulsive, and oppositional traits than TD. Besides, both unaffected siblings of AS probands and unaffected siblings of autism proband groups had more severe oppositional trait, and only the former group had greater hyperactive/impulsive trait than TD. As to CCPT performance, youths with autism had poorer focused attention, more commission errors, and longer RT than their unaffected siblings and TD [25, 27]. Moreover, youths with AS performed worse than TD in focused attention, sustained attention, and vigilance [24, 26, 27] without a statistical difference from their unaffected siblings, who performed in the intermediate position between AS probands and TD. However, when autism and AS were lumped together to compare ASD proband, unaffected siblings, and TD, the significance of comparisons disappeared except for a longer RT in unaffected siblings than in TD. Also, ADHD-related traits were predicted by different correlates of autistic traits and attention profiles for unaffected siblings of autism probands and unaffected siblings of AS probands, with a better model fitting in the latter group.

We found that oppositional trait is the only increased symptom in unaffected siblings of autism (compared to TD) after Bonferroni correction, in line with an earlier study showing that unaffected siblings of autism probands may have more delinquent behaviors but not inattentive problems [63]. Our findings of more ADHD and oppositional traits in unaffected siblings of AS probands than in TD suggest that these traits might be a broader phenotype for AS, implying that siblings of ASDs may need particular attention for their oppositional behaviors as well as ADHD symptoms. Evidence has shown a familial transmission of ADHD-related disorders, with moderate to high heritability for ADHD (0.82) and ODD (0.61) [73]. The shared ADHD-related phenotype between the sib-pairs could be partly explained by the shared genetic backgrounds between the probands and their unaffected siblings. Higher hyperactive/impulsive, oppositional traits in unaffected siblings of AS probands may suggest an overlapping phenomenon or co-segregation of ADHD and autistic traits in the family of AS. Furthermore, our findings of relatively more severe ADHD-related symptoms in AS probands rather than autism probands suggest that these two subtypes may not be the same in their clinical expression regarding ADHD symptoms. These findings wait to be replicated in other independent samples.

As to CCPT performance, our results do not support attention deficits in unaffected siblings of autism probands but show a trend of mild but pervasive attention deficits in unaffected siblings of AS probands, whose performance was at the intermediate position between AS probands and TD without a substantial difference from AS probands. Hence, our findings imply that the compromised focused attention (omission, hit RT SE, and variability) and sustained attention (hit SE block change) assessed by CCPT might be potential endophenotypes for AS but not for autism. Our findings again reflect the differences between unaffected siblings of autism probands and unaffected siblings of AS probands regarding the patterns of attention deficits. Literature has documented attention characteristics in AS, including greater variability as assessed by the CPT [24] or Test of Variables of Attention [25, 26], another test for sustained attention that uses geometric shapes, rather than “X,” as targets. Our recent work also showed that youths with AS had worse focused attention, cognitive impulsivity, sustained attention, and vigilance than controls [27]. This sib-pair study provides further evidence showing that the unaffected siblings of AS probands, like their probands, may have deviant CCPT performance but less in degree, displaying a pattern of compromised focused attention and sustained attention. This contrast might suggest that the unaffected sib-pair of AS may share more attention deficits than autism sib-pairs. Notably, these differences between unaffected siblings of autism and AS will be overlooked when the two conditions are grouped into one single category of ASD, supporting the importance of looking into the subgroups within the whole spectrum concerning the neurocognitive function [45]. In addition, our findings added to the current knowledge of the shared neurocognitive deficits between ASD probands and their unaffected siblings (e.g., executive function such as cognitive flexibility [43–45] and planning [43, 47, 48]) by showing that a more fundamental function, attention performance (e.g., focused attention and sustained attention), may also be involved, particularly for siblings of AS probands. Whether the attention deficits co-segregate with other neurocognitive functions (like the co-segregation of social cognition and executive function [74]) and how these deficits (e.g., higher response variability, lower vigilance) influence other neurocognitive functions (e.g., cognitive flexibility) in the siblings warrant further investigation.

Our findings of positive associations between stereotyped behaviors/interest and ADHD-related traits in unaffected siblings correspond to those found in probands with ASD [13, 66]. Such correlation can be explained by that child with ASD displaying high degrees of stereotypes cannot be attentive to other focuses in the surroundings [13]. Combining previous findings in ASD probands [13, 66] and new findings in unaffected siblings, stereotyped behaviors seem to be associated with ADHD-related traits, implying the co-segregation between the two traits in the family of ASD.

Except for stereotyped behaviors, unaffected siblings of autism probands and unaffected siblings of AS probands showed distinct correlates for their ADHD-related traits. First, the association between hyperactivity and impaired communication in unaffected siblings of AS probands was consistent with previous studies in ASD [13, 66] and ADHD [75]; likewise, the negative association between age and hyperactive/impulsive trait was similar to previous findings (in ASD [12, 66] or ADHD [76–78]). On the other hand, the positive associations between perseveration and both inattentive and hyperactive/impulsive traits suggest that ADHD traits in unaffected siblings of autism probands might be associated with the problems of attention flexibility. Although perseveration (lack of flexibility) is not recognized as one of ADHD symptoms [79], a recent study suggests an overlapping between perseveration and hyperactivity phenotypes in a mouse model (Xp22.3 deletion) for neurodevelopmental disorders [80], providing a potential biological basis for our findings. By contrast, the significant correlates of inattentive and oppositional traits in unaffected siblings of AS probands (i.e., detectability and response style) corresponded to the accuracy problems and risky response style reported in ADHD [55, 81, 82]. In summary, ADHD-related traits in unaffected siblings of ASD probands can be attributable to autistic traits and CCPT performance, with distinct components in siblings of autism and siblings of AS and a better model fitting in the latter group (unaffected siblings of AS probands). Our findings provide the evidence to support the following points. First, unaffected siblings of AS probands might demonstrate similar attention patterns to youths with ADHD reported in the literature [55, 81, 82]. Second, the specific relationships between ADHD symptoms and ASD symptoms in the “unaffected siblings” of ASD echoed a recent study showing genetic influences between dimensional ASD and ADHD symptoms during child and adolescent development [83]. Lastly, our results further suggested that these associations may vary within the autism spectrum.

Our finding that unaffected siblings of AS probands performed in between probands with AS and TD on several aspects of attention assessed by CCPT implies that these traits may constitute a substantial endophenotype of AS, and could be considered as one of the surrogates in searching genetic etiology of AS. However, whether the observed CCPT deficits were inherent to ASD or only a reflection of co-occurring ADHD traits needs further clarification. In the subsidiary analysis, we found the CCPT deficits in the AS probands disappeared when attention problems were controlled while those in autism probands remained significant (data not shown), implying that co-occurring ADHD traits may contribute to the CCPT deficits in AS but not autism. Although the unaffected siblings did not show more inattentive symptoms, CCPT deficits underlying AS and ADHD might suggest their utility in searching for common genetic etiology underlying the overlapping neurodevelopmental conditions, like Rommelse et al. [84] proposed. Family and twin studies support the hypothesis that ASD and ADHD may originate from similar familial/genetic factors [85], with evidence showing that the probability of the co-twins of ASD probands having a diagnosis of ADHD was 44% in monozygotic co-twins versus 15% in dizygotic co-twins [86]. It was estimated that around 50–72% of the contributing genetic factors overlap between ASD and ADHD [87]. With frequent overlappings between neurodevelopmental conditions, a dimensional approach breaking down the diagnosis boundary yet focusing on common endophenotypes, like CPT deficits, should be considered in future genetic research in neurodevelopmental disorders with ASD or ADHD traits. Using a sib-pair design, we demonstrated that the phenotype of AS might reveal more ADHD-related traits and a trend for subtle but pervasive attention deficits assessed by CCPT, in support of a previous hypothesis that AS may be a mixed syndrome with overlapping autistic symptoms and ADHD-related traits [26]. Taken together, our findings may shed light on a shared genetic backgrounds between ASD and ADHD, with yet different genetic components between autism and AS (to some extent), echoing a recent report suggesting shared risk genes but the different prevalence of *SHANK3* variants between the subtypes of ASD [88].

This study has four limitations. First, the probands and TD were male-predominant and age-limited whereas the siblings were equal in sex distribution. Because ASD population is male predominant, this problem cannot be avoided in any study design. Therefore, we adjusted sex and age in all statistical analyses. Second, we used parents’ reports to evaluate inattentive and hyperactive/impulsive symptoms but not the clinical diagnosis. Though, SNAP-IV is a valid instrument to measure ADHD and oppositional symptoms dimensionally; CBCL attention problem subscore was also collected to capture the behavior phenotype by checklist. Both measures consistently showed increased ADHD traits in the probands, with SNAP-IV more specifically reflected the hyperactive/impulsive trait in the siblings of AS probands. Third, this study design is unable to answer whether the CCPT deficits in ASD probands or siblings were specific to ASD or were associated with the comorbid ADHD. To address the specificity of CCPT deficits, future studies may consider comparing CCPT performance either among “ASD only,” “ASD comorbid with ADHD,” “ADHD only,” and “controls without ADHD and ASD;” or between unaffected siblings “with ADHD,” and “without ADHD.” Finally, IQ was not controlled in the group comparisons because the performance IQ of unaffected siblings was not statistically different from TD. However, lower IQ in the probands, which was particularly true in probands with autism, may cause the group comparisons confounded by IQ. Though, researchers have argued that controlling IQ may be over-adjustment and may not be necessary since the deviations on intelligence could be inherent to ASD psychopathology. To ensure that all the participants understand the task, participants who had full-scale IQ below 70 were excluded. Such restriction of the sample may limit the generalizability of our results to the whole ASD population. Nevertheless, several features of this study constitute its strengths. This study provides important evidence regarding the hyperactive/impulsive and oppositional traits in unaffected siblings of ASD probands, in a representative sample recruited from both clinical population and community. In addition, an objective, valid, and widely used instrument, CCPT, was used to assess and to compare a wide range of attention performance among the ASD probands, unaffected siblings, and TD groups, based on a newly proposed factor structure [61].

## Conclusions

In conclusion, based on a large-scale sib-pair sample, our findings provided evidence to support that compromised focused attention and sustained attention might serve as potential endophenotypes for genetic studies in AS. Attention performance on CCPT may be used in detecting genetic effects underlying complex cognitive functions mediated by a broad functional brain network. The unaffected siblings of probands with ASDs usually obtain less attention from the parents and clinicians than their probands. However, they may benefit from clinical assessment and management for co-occurring hyperactive/impulsive and oppositional traits, particularly for the siblings of AS probands.

## Additional files

Additional file 1: Autistic symptoms of probands (autism and Asperger’s disorder), unaffected siblings and typically developing controls. This table presents five group comparison on Social Communication Questionnaire subscores and the subcores of Autism Diagnostic Interview-Revised for the probands. (PDF 174 kb)

Additional file 2: Comparison of ADHD-related symptoms and CCPT performance between probands with autism spectrum disorders, unaffected siblings of ASD, and typically developing controls. This table presents the three group comparison of ADHD-related symptoms and CCPT performance between probands with autism spectrum disorders, unaffected siblings of ASD, and typically developing controls adjusting for sex and age. (PDF 162 kb)

Additional file 3: Comparison of CCPT performance between probands with autism, unaffected siblings of autism probands, and typically developing controls. Comparison of CCPT performance between probands with autism, unaffected siblings of autism probands, and typically developing controls adjusting for sex and age. (PDF 103 kb)

Additional file 4: Selected Conners’ Continuous Performance Test indexes showing that probands with Asperger’s disorder (AS) performed differently from typically developing youths (TD) and their unaffected siblings performed in the intermediate position. (a) Omission, Reaction time standard errors (RT SE), and Variability; (b) Reaction time; (c) Hit reaction time standard error (Hit SE) block change and Hit reaction time inter-stimulus (Hit RT ISI) change (Note. The index of Hit RT ISI didn’t pass False Discorvery Rate correction.) (PDF 79 kb)

## Abbreviations

ADHD: Attention-deficit/hyperactivity disorder
ADI-R: Autism Diagnostic Interview-Revised
AS: Asperger syndrome
ASD: Autism spectrum disorder
CBCL: The Child Behavior Checklist
CCPT: The Conners’ Continuous Performance Test
CPT: Continuous Performance Test
ISI: Inter-stimulus interval
RT: Reaction time
SCQ: The Social Communication Questionnaire
SE: Standard errors
SNAP-IV: The Swanson, Nolan, and Pelham, version IV scale
TD: Typically developing youths
US: Unaffected siblings
