# Supplementary material for: ADHD-related symptoms and attention profiles in the unaffected siblings of probands with autism spectrum disorder: focus on the subtypes of autism and Asperger’s disorder
Source: Mol Autism. 2017 Jul 25;8:37. doi: 10.1186/s13229-017-0153-9 (PMC5526322; doi:10.1186/s13229-017-0153-9)
Supplement: Supplementary file 1 — Autistic symptoms of probands (autism and Asperger’s disorder), unaffected siblings and typically developing controls. This table presents five group comparison on Social Communication Questionnaire subscores and the subcores of Autism Diagnostic Interview-Revised for the probands. (PDF 174 kb) [file 13229_2017_153_MOESM1_ESM.pdf]

Additional file 1. Autistic symptoms of probands (autism and Asperger's disorder (AS)), unaffected siblings (US) and typically-developing controls (TD)

Group	Autism (*n* =122)	AS (*n* = 77)	US of autism (*n* = 122)	US of AS (*n* = 77)	TD (*n* = 196)	F	*P*
**Social Communication Questionnaire**							
Social deficits	11.46±5.55	10.50±5.53	1.92±2.66	2.69±2.57	2.53±2.62	127.80	<.001
Communication deficits	4.45±2.13	3.82±1.81	0.70±1.10	0.88±1.43	0.61±1.09	138.83	<.001
Stereotyped behaviors	5.51±2.73	4.64±2.71	0.89±1.28	1.38±1.43	0.89±1.42	107.00	<.001
Total scores	20.16±7.23	17.86±7.23	3.30±3.53	4.74±4.40	3.80±3.53	219.98	<.001
**Autism Diagnostic Interview- Revised (ADI-R)**							
**Current:**							
A: Social deficits	10.28±4.94	10.05±4.35				0.11	0.742
B: Communication (verbal)	11.81±4.62	11.14±4.16				0.00	0.984
B: Communication (non-verbal)	5.64±3.21	5.73±2.53				0.34	0.564
C: Restricted interests and stereotyped behaviors	5.36±2.67	5.36±2.68				0.01	0.909

**Severe:**						
A: Social deficits	22.52±5.31	18.69±6.70			19.76	<.001
B: Communication (verbal)	16.55±3.69	13.55±4.33			27.05	<.001
B: Communication (non-verbal)	8.51±2.73	7.00±3.12			12.83	<.001
C: Restricted interests and stereotyped behaviors	7.32±2.38	6.65±2.80			3.22	0.074

Note: Only a portion of probands with autism spectrum disorders have ADIR B subscores (N=47 in probands with autism, N=22 in probands with Asperger's disorder). Post hoc analysis showed that autism probands showed more autistic traits than their unaffected siblings and TD in each autistic traits (social deficits, communication deficits, and stereotyped behaviors). Similarly, AS probands showed more autistic traits than their unaffected siblings and TD. AS=Asperger's disorder; TD=typically-developing controls; US=unaffected siblings
