# Supplementary material for: ADHD-related symptoms and attention profiles in the unaffected siblings of probands with autism spectrum disorder: focus on the subtypes of autism and Asperger’s disorder
Source: Mol Autism. 2017 Jul 25;8:37. doi: 10.1186/s13229-017-0153-9 (PMC5526322; doi:10.1186/s13229-017-0153-9)
Supplement: Supplementary file 2 — Comparison of ADHD-related symptoms and CCPT performance between probands with autism spectrum disorders, unaffected siblings of ASD, and typically developing controls. This table presents the three group comparison of ADHD-related symptoms and CCPT performance between probands with autism spectrum disorders, unaffected siblings of ASD, and typically developing controls adjusting for sex and age. (PDF 162 kb) [file 13229_2017_153_MOESM2_ESM.pdf]

Additional file 2. Comparison of ADHD-related symptoms and CCPT performance between probands with autism spectrum disorders, unaffected siblings of ASD, and typically-developing controls

Group	ASD (n =199)	US (n = 199)	TD (n = 196)	F	*P*	Comparison#	*Cohen's d* ASD: TD	*Cohen's d* US: TD	*Cohen's d* ASD: US
**SNAP-IV**									
Inattentive	15.81±6.57	6.43±6.00	5.46±4.65	140.52	<.0001	ASD>US,TD	1.82	0.18	1.49
Hyperactive/impulsive	11.07±6.99	4.10±4.95	2.92±3.93	93.49	<.0001	ASD>US,TD	1.44	0.26	1.15
Oppositional	9.62±6.12	6.24±5.26	3.60±3.89	49.19	<.0001	ASD>US>TD	1.17	0.57	0.59
**Focused attention**									
Omission	16.84±27.80	7.62±8.72	6.35±7.41	22.82	<.0001	ASD>US,TD			
RT SE	435.98±142.90	407.51±92.08	381.04±67.13	28.33	<.0001	ASD>US,TD			
Variability	13.79±11.11	9.79±6.06	8.63±5.50	16.63	<.0001	ASD>US,TD			
Perseveration	13.93±17.66	7.73±13.12	7.02±14.31	11.55	<.0001	ASD>US,TD			
Detectability	0.33±0.39	0.47±0.38	0.43±0.36	6.62	0.0014	ASD<US,TD			

**Cognitive impulsivity**						
Commission	23.51±8.83	19.94±8.42	20.85±8.31	8.01	0.0004	ASD>US,TD
Reaction time	435.98±142.90	407.51±92.08	381.04±67.13	20.51	<.0001	ASD>US>TD
Response style	0.94±2.64	1.65±9.62	0.58±1.25	1.68	0.1873	-
**Sustained attention**						
Hit RT block change	0.01±0.05	0.01±0.03	0.01±0.03	1.36	0.2564	-
Hit SE block change	0.08±0.12	0.07±0.11	0.04±0.09	4.51	0.0113	ASD>TD
**Vigilance**						
Hit RT ISI change	0.09±0.08	0.08±0.05	0.08±0.04	4.36	0.0132	ASD>US,TD
Hit SE ISI change	0.12±0.19	0.11±0.16	0.10±0.16	1	0.3667	-

Bonferroni correction $p < 0.05$

Abbreviation: ASD=autism spectrum disorders; ISI=inter-stimulus interval; RT=Reaction time; SE=Standard error; TD=typically-developing controls; US=unaffected siblings of ASD
