# Supplementary material for: ADHD-related symptoms and attention profiles in the unaffected siblings of probands with autism spectrum disorder: focus on the subtypes of autism and Asperger’s disorder
Source: Mol Autism. 2017 Jul 25;8:37. doi: 10.1186/s13229-017-0153-9 (PMC5526322; doi:10.1186/s13229-017-0153-9)
Supplement: Supplementary file 3 — Comparison of CCPT performance between probands with autism, unaffected siblings of autism probands, and typically developing controls. Comparison of CCPT performance between probands with autism, unaffected siblings of autism probands, and typically developing controls adjusting for sex and age. (PDF 103 kb) [file 13229_2017_153_MOESM3_ESM.pdf]

Additional file 3. Comparison of CCPT performance between probands with autism, unaffected siblings of autism probands, and typically-developing controls

Group	Autism (n =122)	US of autism (n = 122)	TD (n = 196)	F	*P*	Comparison#
**Focused attention**						
Omission	20.68±31.56	7.16±8.42	6.35±7.41	27.80	<.001	A>US,TD
RT SE	15.71±12.55	9.59±5.72	8.63±5.50	33.05	<.001	A>US,TD
Variability	29.84±25.92	18.22±15.70	16.89±16.54	17.96	<.001	A>US,TD
Perseveration	16.89±19.19	6.73±10.34	7.02±14.31	15.56	<.001	A>US,TD
Detectability	0.30±0.40	0.48±0.40	0.43±0.36	5.88	0.004	A<US,TD
**Cognitive impulsivity**						
Commission	24.32±8.94	19.39±8.55	20.85±8.31	8.00	<.001	A>US,TD
Reaction time	461.20±163.47	412.46±96.85	381.04±67.13	24.49	<.001	A>US,TD
Response style	1.16±3.35	2.35±12.26	0.58±1.25	2.54	0.083	-

**Sustained attention**						
Hit RT block change	0.01±0.05	0.01±0.03	0.01±0.03	0.43	0.652	-
Hit SE block change	0.07±0.12	0.07±0.10	0.04±0.09	3.39	0.037	-
**Vigilance**						
Hit RT ISI change	0.09±0.09	0.08±0.05	0.08±0.04	2.21	0.114	-
Hit SE ISI change	0.12±0.19	0.11±0.16	0.10±0.16	0.23	0.793	-

Bonferroni correction $p < 0.05$

Abbreviation: ISI=inter-stimulus interval; RT=Reaction time; SE=Standard error; TD=typically-developing controls; US=unaffected siblings of autism probands
