# Supplementary material for: ADHD-related symptoms and attention profiles in the unaffected siblings of probands with autism spectrum disorder: focus on the subtypes of autism and Asperger’s disorder
Source: Mol Autism. 2017 Jul 25;8:37. doi: 10.1186/s13229-017-0153-9 (PMC5526322; doi:10.1186/s13229-017-0153-9)
Supplement: Supplementary file 4 — Selected Conners’ Continuous Performance Test indexes showing that probands with Asperger’s disorder (AS) performed differently from typically developing youths (TD) and their unaffected siblings performed in the intermediate position. (a) Omission, Reaction time standard errors (RT SE), and Variability; (b) Reaction time; (c) Hit reaction time standard error (Hit SE) block change and Hit reaction time inter-stimulus (Hit RT ISI) change (Note. The index of Hit RT ISI didn’t pass False Discorvery Rate correction.) (PDF 79 kb) [file 13229_2017_153_MOESM4_ESM.pdf]

Supplementary Figure 1. Selected Conners' Continuous Performance Test indexes showing that probands with Asperger's disorder (AS) performed differently from typically-developing youths (TD) and their unaffected siblings performed in the intermediate position: (a) Omission, Reaction time standard errors (RT SE), and Variability; (b) Reaction time; (c) Hit reaction time standard error (Hit SE) block change and Hit reaction time inter-stimulus (Hit RT ISI) change (Note. The index of Hit RT ISI didn't pass False Discovery Rate correction.)

(a)

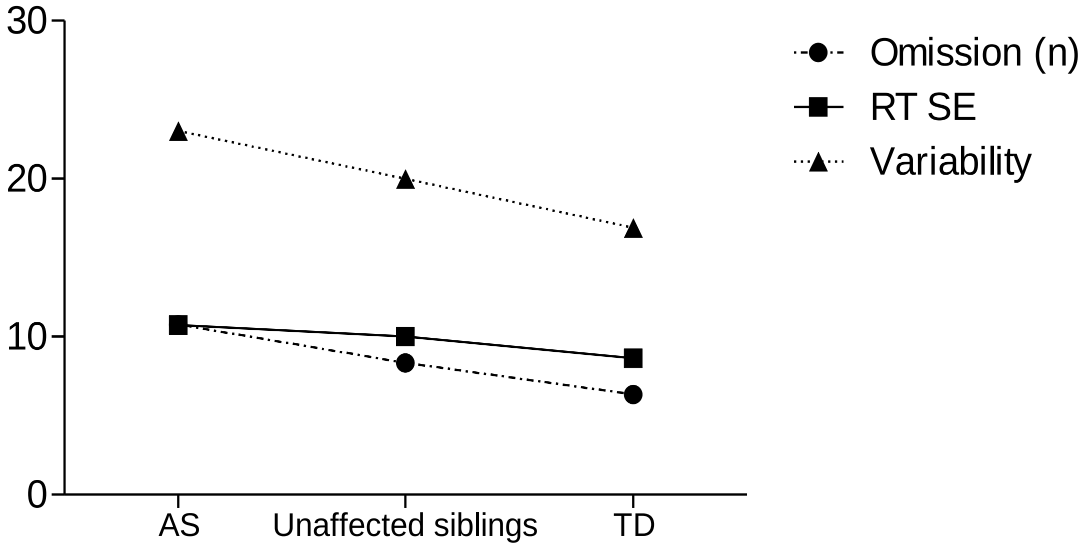
30
20
10
0
AS
Unaffected siblings
TD
Omission (n)
RT SE
Variability

(b)

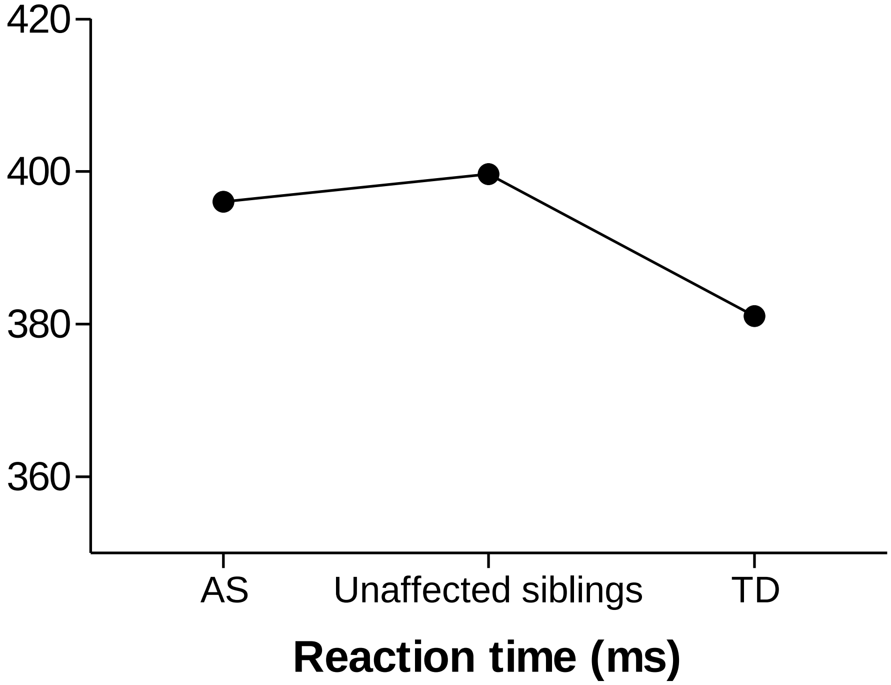

(c)

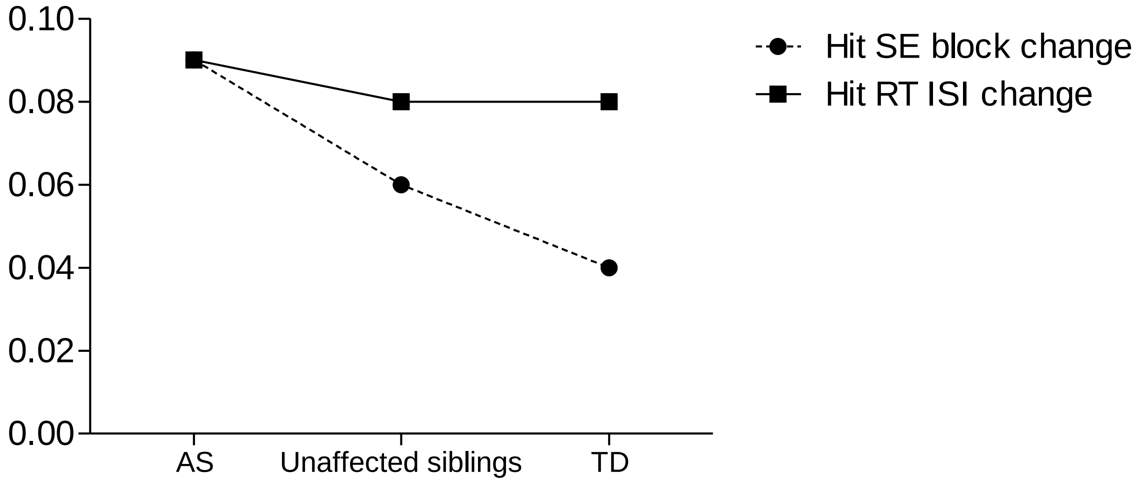
0.10
0.08
0.06
0.04
0.02
0.00
AS
Unaffected siblings
TD
Hit SE block change
Hit RT ISI change
